# The Role of TNF-α in the Pathogenesis of Idiopathic Nephrotic Syndrome and Its Usefulness as a Marker of the Disease Course

**DOI:** 10.3390/jcm13071888

**Published:** 2024-03-25

**Authors:** Agnieszka Pukajło-Marczyk, Danuta Zwolińska

**Affiliations:** Department of Pediatric Nephrology, Wroclaw Medical University, Borowska 213, 50-556 Wroclaw, Poland; danuta.zwolinska@umw.edu.pl

**Keywords:** tumor necrosis factor alpha, idiopathic nephrotic syndrome, proteinuria, minimal change disease, children

## Abstract

**Background**: The pathogenesis of idiopathic nephrotic syndrome (INS) has not been fully explained. Among the likely factors, tumor necrosis factor - alpha (TNF-α) is considered. We aimed to evaluate the TNF-α (sTNF-α, uTNF-α) levels in the serum and urine of INS children, with the aim of determining its association with proteinuria, and of determining its usefulness as a marker of the disease severity. **Methods**: Fifty-one examined patients were divided into subgroups depending on the number of relapses as follows: group IA—first episode; group IB—more than two relapses, and according to treatment modality; group IIA—glucocorticosteroids (GS) alone; and group IIB—GS with immunosuppressants. Healthy age-matched children served as the control group. **Results**: sTNF-α and uTNF-α levels were significantly increased in active phases in the whole INS group compared to the control group. They decreased in remission, but remained significantly higher when compared to the control group. During remission in the IB group, sTNF-α levels were significantly higher than in IA, whereas, in the relapse phase, these values were similar. In the IA group, a positive correlation between proteinuria and sTNF-α was demonstrated. **Conclusions**: Our findings suggest that TNF-α plays a role in the development of INS, and may be used as a prognostic marker, as well as an indicator for the continuation of therapy. Additional research is required to verify this statement.

## 1. Introduction

Idiopathic nephrotic syndrome (INS) is the most common form of glomerulopathy among children, representing more than 90 percent of cases in children between 1 and 10 years of age. It is characterized by massive proteinuria associated with hypoalbuminemia, dyslipidemia, and edema as a result of the increased permeability of the glomerular barrier and podocyte foot process effacement. The morphological findings of INS mainly include minimal change disease (MCD), and less often include focal segmental glomerulosclerosis (FSGS) or mesangial proliferation. INS occurs with periods of relapse and remission [1,2]. Depending on the response to steroids, INS is classified as steroid sensitive (in most cases), steroid dependent, or steroid resistant, where the use of other immunosuppressive drugs is required [3,4].

Despite many studies, the pathogenesis of INS remains unclear. Undoubtedly, the immune system dysregulation plays a crucial role. The first hypothesis pointed to a circulating permeability factor from dysfunctional T-cells, and, among many candidates, several cytokines have been considered, including TNF-α [5,6,7,8,9]. 

Further studies have shown that the disturbances in the ratio of T-cell subpopulations, as well as B-cells, T-regulatory cells (Tregs), bacterial and viral antigens, CD 80, and CTLA-4 molecules, contribute to the development of INS. The two-hit theory combines these elements, emphasizing that the pathogenesis of INS is a complex interaction between soluble circulating factors, immunocompetent cells, podocytes, and a multi-stage control system [10,11]. In the development of INS caused by FSGS, the impact of the soluble urokinase plasminogen activator receptor (suPAR) and its influence on glucocorticoid receptors, which are responsible for the effectiveness of steroid therapy, are also taken into account [12,13,14].

There is still significance in understanding the role of circulating factors such as TNF-α in the development of proteinuria in INS; however, so far, only a few inconclusive studies have been published regarding this topic [15,16,17,18,19,20]. 

TNF-α is a pleiotropic and proinflammatory cytokine, produced mainly by activated macrophages and monocytes as a prohormone, constituting 233 amino acids. This transmembrane protein (mTNF-α) is expressed on the cell surface, where it either continues to reside or is actively cleaved by TNF-converting enzymes. The resulting soluble form of the TNF (sTNF-α) of 17 kDa, composed of 157 soluble amino acids, is detectable in plasma. Both mTNF-α and sTNF-α have cellular functions that are mediated by one of two receptors: either TNFR1, expressed in all human tissues, or TNFR2, expressed mainly in immune cells, neurons, and endothelial cells. Upon binding to receptors, signal transduction pathways are initiated [21,22].

TNF-α is also secreted by antigen-presenting cells and by mesangial cells, as well as kidney epithelial cells. Factors such as interferon-gamma, lipopolysaccharides of bacterial walls, and platelet-derived growth factors increased TNF-α synthesis. It may also be produced by the autocrine pathway. TNF-α was originally perceived as a factor which leads to tumor necrosis; currently, however, it is recognized as a multifunctional cytokine, involved in various cell functions, such as differentiation, proliferation, inflammation, and immune controls. Physiologically, this cytokine plays an important role in the immune response; however, the overproduction can be harmful, leading to chronic inflammation and the development of various immune-mediated diseases, particularly autoimmune diseases [23,24,25]. The involvement of TNF-α in the pathogenesis of autoimmune diseases is somewhat confirmed by the effective responses to anti-TNF-α antibody therapy [26,27].

Some experimental studies confirm the involvement of TNF-α in glomerular damage in various types of glomerulopathy [28,29,30,31]. Convincing data on this topic were presented by Gomez-Chiarri et al., who studied MCD nephrosis, induced in rats by adriamycin or puro-mycin aminonucleoside. They showed an increase in TNF-α synthesis by mesangial cells, glomerular epithelial cells, and tissue-infiltrating macrophages and monocytes simultaously, demonstrating that proteinuria reached maximal levels, as well as the highest level of TNF-α being observed [32].

Therefore, the goal of our study was to assess sTNF- α and uTNF- α levels in INS children, and to determine its pathogenic role in the development of nephrotic range proteinuria, as well as to assess the usefulness of TNF- α as a predictor of the disease course.

## 2. Materials and Methods 

A total of 69 children were enrolled in the study, including 51 children with INS and 18 healthy peers. The analyzed group was the same as the group used in our previous study concerning INS [19]. The group of patients, consisting of 19 girls and 32 boys, aged 1.25–18 years (mean age 8.86 ± 5.2 years), was divided into subgroups according to the number of disease relapses, and depending on the treatment used. Group IA included 20 children presenting their first episode of INS (5 girls, 15 boys; mean age 5.90 ± 4.91 years), while group IB consisted of 31 children (14 girls, 17 boys; mean age 10.31 ± 4.81 years old), who had experienced 2–16 relapses (average number of relapses—11.8). In order to analyze the applied treatment, the group of patients was divided into group IIA—children treated with GS alone (26 children, including 7 girls, 19 boys; mean age 5.59 ± 4.04 years) and group IIB—patients requiring additional immunosuppressive drugs (22 children, including 9 girls, 13 boys; mean age 12.31 ± 4.06 years). The therapies used included cyclosporine (CyA), mycophenolate mofetil (MMF), and azathioprine (AZA). The distribution of immunosuppressive drugs administered in group IIB was as follows: 17 children—CyA + GS, 2 children—MMF + GS, 2 children—CyA + AZA + GS, 1 child—CyA + MMF + GS. In all children, if nephrotic syndrome was diagnosed or relapse occurred, a standard dose prednisone (2 mg/kg/day) was administered. Some children required pulses of methylprednisolone at a dosage of 0.5 g/dose. The group of 18 healthy subjects (12 girls and 6 boys, with a mean age of 8.40 ± 3.87 years) served as controls. These children had been diagnosed with primary nocturnal enuresis, or had suspected urinary tract abnormalities that were ultimately not confirmed. 

The study examined changes in TNF-α concentration during relapse and remission in a specific patient, aiming to evaluate the dynamics of this factor within two phases of the same disease episode. The diagnosis of INS was established based on the International Study of Kidney Disease in Children (ISKDC) criteria [33]. Remission was defined as the absence of proteinuria for at least three consecutive days. Relapse was denoted by a reappearance of proteinuria for three consecutive days. In each patient, blood samples were collected in the morning, after an overnight fast, and during scheduled routine laboratory tests. The samples were allowed to clot for 30 minutes, and then centrifuged at room temperature for 15 minutes. On the same day, the first morning urine samples were collected. The obtained material was centrifuged for 15 minutes, and then the sediment was removed. The collected samples of the biological material were frozen at −70 °C and stored until assayed. The concentration of TNF-α in blood and urine was determined in each patient. Additionally, creatinine, albumin, total cholesterol, and CRP in blood samples were determined, and the creatinine and protein levels in urine were also verified. In the examined children, the parameters of kidney function and inflammation were within normal limits. Creatinine concentration was determined using the enzymatic method, and the Schwartz formula was used to calculate the estimated glomerular filtration rate (eGFR) [34]. Proteinuria was assessed based on the ratio of protein and creatinine concentrations in urine (uPCR), and the value defining nephrotic proteinuria was uPCR > 2 (2 mg/mg) [2]. Other tests were determined by standard methods using the Olympus 5800 analyzer.

The sTNF-α and uTNF-α concentrations were determined using the ELISA immunoenzymatic method and Quantikine R&D System kits (catalog number: DTA00C), according to the manufacturer’s instructions. The tests were carried out twice, and then the average of the obtained results was taken. TNF-αvalues are expressed in pg/mL. The sensitivity of the method for determinations is within the range of 0.5–5.5 pg/mL; the average values were 1.6 pg/mL for both sTNF-α and uTNF-α. 

The study was conducted according to the Declaration of Helsinki guidelines, and was approved by the Bioethics Committee of the Medical University of Wrocław (no. KB-199/2009). The parents of the children included in the study and participants over 16 years old provided their informed consent to participate in this project.

The results of our studies were expressed as median values and interquartile ranges. Taking into account the small number of patients in each group, the non-parametric Kruskal–Wallis rank sum test was used to verify the hypothesis regarding the equality of median values in terms of the studied parameters. The verification of the hypothesis regarding the equality of the median values in the range of parameters tested in individual dependent samples (e.g., relapse and remission) was carried out using the non-parametric Wilcoxon paired sequence test. Pearson`s correlation coefficient r was used to assess the relationship between the studied parameters. A value of *p* < 0.05 was considered statistically significant. Statistical analysis was performed using the EPIINFO Ver. software package, 7.1.1.14 Centers for Disease Control and Prevention (CDC), Atlanta, GA, USA (as of 2 July 2013). 

## 3. Results

All parameters in the controls were within the normal limits. 

During INS relapse in children, those requiring additional immunosuppressive treatment had significantly higher levels of proteinuria when compared to the group treated with GS alone, based on our analysis of biochemical parameters. Table 1 displays the data of basic biochemical parameters in children with INS across various groups. 

The sTNF-α and uTNF-α levels were significantly higher in the INS group during both relapse and remission periods when compared to the control group. The levels of TNF-α were higher at the relapse phase when compared to remission, both in the serum and urine (Table 2). 

Compared to the first episode of INS, significantly higher sTNF-α levels were found in the remission group, but not in the relapse group of INS patients with subsequent relapses. However, urine TNF-α levels were comparable in both subgroups, and did not depend on the stage of the disease (Table 3).

When analyzing the effect of immunosuppressive drugs on serum and urine TNF-α in groups IA and IB, no significant differences were found in either the relapse or remission groups (Table 4). 

In the relapse subgroup of INS children, no correlation was found between the serum and urine TNF-α and CRP, albumin, total cholesterol, and proteinuria. When analyzing similar data in individual subgroups, the only positive correlation between proteinuria and sTNF-α in the relapse subgroup of the IA group was found (r = 0.44, *p* < 0.05).

## 4. Discussion

It has long been suggested that TNF-α is one of the circulatory permeability factors involved in the pathogenesis of nephrotic syndrome. However, the published results of previous studies have been discordant [35,36]. Our study has shown significantly higher sTNF-α and uTNF-α levels in the relapse subgroup of the whole INS group compared to the controls. Following remission, these values decreased, but they were still significantly higher than those in the control group. These results are consistent with those obtained by Lama et al. and Bustos et al. [37,38]. regarding the value of sTNF-α, this cytokine was not tested in urine. Additionally, the Spanish authors assessed TNF-α mRNA, IL-1β, and IL-6 expressions in peripheral blood mononuclears, as well as in their synthesis in the cell culture supernatant. In both cases, when compared to the remission and control subgroups, a significant increase in TNF-α mRNA in the relapse subgroup was found. They did not observe such differences in relation to IL-1β and IL-6, suggesting that only TNF-α may play an important role in the pathogenesis of nephrotic proteinuria [38]. 

In turn, Weissach et al. [39], in their pilot study involving children with steroid-sensitive and steroid-resistant nephrotic syndrome, showed a significant increase in sTNF-α in both groups when compared to the control, and significantly higher values in steroid-resistant INS patients. Moreover, the mean sTNF-α levels in this group were similar both before and after treatment, which, according to the authors, strengthens the suggestion of the impact of TNF-α on the glomerular permeability of the vascular wall. Recently, Roca et al. [40] published the results of an observational study regarding the activation of the acute inflammatory phase response in INS, and the association with clinicopathological phenotypes with response to corticosteroids. They also showed a significant increase in sTNF-α levels in 101 patients (20% of whom were children) when compared to controls, as well as other inflammatory proteins including IL-6, suPAR, and hemopexin. However, no correlation with proteinuria, serum albumin, and eGFR was found, which proves the complexity of the pathogenesis of INS. It should be emphasized that the study group was relatively large, although it mainly consisted of adults, and the tests were performed during the relapse of INS before steroids were administered. However, not all studies have demonstrated an increase in sTNF-α [35,41].

In turn, Cho et al. [35], when examining TNF-α and IL-8 in 19 children with NS due to MCD, observed only an increase in the uTNF-α level in the relapse subgroup when compared to the remission and control subgroups. This is consistent with the results of our study. It should be added that significantly lower values in the remission subgroup compared to those in the relapse subgroup were observed; however, these remained significantly higher than in healthy children. Korean researchers also assessed the direct impact of TNF-α and IL-8 on glomerular epithelial cells, which are widely considered to be targets in the pathogenesis of MCD. Using the Millicell system, they found no direct effect on heparan sulfate proteoglycan gene expression, nor the synthesis of heparan sulfate in GECs (glomerular epithelial cells). However, they found that the serum of patients with nephrotic proteinuria led to significant albumin loss by GECs, concluding that, although TNF-α does not play a specific pathogenetic role in MCD, its indirect impact cannot be excluded.

Laflam et al. [36] reached similar conclusions after conducting experimental studies concerning rats with induced MCD lesions. A five-day supply of TNF-α into the renal artery showed a significant increase in albuminuria on days four and five following the end of the infusion. Contrary to expectations, no differences in TNF-α values were observed between remission and relapse. Similarly to Cho et al., the authors suggest that, although TNF-α does not play a direct role in the development of nephrotic proteinuria, its influence on the severity of albuminuria is possible via a paracrine pathway.

The aim of our study was also to verify the hypothesis that TNF- α may differentiate patients experiencing their first onset of INS from those with subsequent relapses. The main finding of this study was that children who experienced subsequent relapses had significantly higher levels of sTNF-α during remission when compared to those who experienced their initial episode of INS. The values of TNF-α in urine and serum were similar in the acute phase of INS in both analyzed groups. This interesting observation suggests a predictive value of sTNF-α in relation to the clinical course of the disease. The above data differ slightly from those described by Rizk et al. [42], who assessed the sTNF-α levels of 60 children with INS, divided into the three following groups: in the first relapse, in the active phase of the first INS recurrence, and in remission. Consistent with our results, they showed a significantly higher sTNF-α level in the first acute episode and first relapse when compared to remission and control groups; our observations regarding the persistence of higher values of this protein in remission compared to healthy peers were also corroborated. However, contrary to our results, they noticed higher sTNF-α concentrations in the first acute episode of INS compared to the relapse of children with subsequent relapses. However, it should be emphasized that our IB group included patients with several recurrences, which could be the reason for these discrepancies. 

When analyzing the results of sTNF-α and uTNF-α, depending on the type of drugs used, we did not observe any differences between the groups, regardless of the phase of the disease. This is consistent with the results obtained by Daniel et al. [41], who studied sTNF-α in two groups of children with INS; these groups were steroid-sensitive and steroid-resistant. Immunosuppressive drugs were also used in this study. In addition, they also analyzed nine different lymphocyte subpopulations, as well as other cytokines produced by monocytes, Th1 and Th2 lymphocytes. Among other findings, they stated that the reduction of the Th1/Th2 lymphocyte ratio in patients with steroid-sensitive INS, both in relapse and remission, was observed. An increase in NK cells (CD16+) in the relapse subgroup compared to the remission subgroup was also confirmed, which indicates an increased cytotoxic potential. Moreover, it was highlighted that the abnormalities were related to adhesion molecules and other cytokines, which is further evidence of the complexity of immunological mechanisms in the pathogenesis of INS. Among these mechanisms, sTNF-α plays a vial role, as confirmed in our study via the positive correlation with proteinuria in the first relapse of INS.

The discrepancy between the study results obtained by the different authors mentioned above could be partially explained by various factors such as the inclusion of patients with different stages of disease activity, the influence of various drugs at the time of measurement, various morphological glomerular changes causing INS, and methodological aspects.

Our study has several limitations that must be highlighted. First, it is a single-center and retrospective study. Second, all group sizes are small, and, due to this fact, we did not analyze the data in terms of the histopathological pattern of INS. Third, the subgroup of INS children receiving combination therapy is rather heterogenic. However, it is challenging to gather a homogeneous group of pediatric patients with steroid-dependent and frequently recurring INS who require alternative treatments. To confirm our observations, particularly regarding TNF-α as a predictor of the disease course, a long-term prospective study would be necessary. 

## 5. Summary

Our results do not provide sufficient evidence to support the direct involvement of TNF-α in the pathogenesis of INS. However, significantly elevated serum and urine TNF-α levels in relapses of INS in children, decreases in remission, and a positive correlation between sTNF-α and proteinuria during the relapse may suggest its role in the development of INS. The maintenance of increased sTNF-α and uTNF-α values after reaching remission speaks to the persistence of immune system activation. Increased sTNF-α levels in patients with subsequent relapses as compared to children experiencing their first onset of INS indicate that sTNF-α may be a useful prognostic marker of the disease course and an indicator for prolonged therapy. Further prospective studies are necessary to confirm our observations.

## Figures and Tables

**Table 1:** Median values and interquartile ranges of selected biochemical parameters in children with INS and the examined subgroups, categorized by relapse number and treatment modality.

	Biochemical Parameters			
Groups	Serum Albumin [g/dL]	Total Cholesterol [mg/dL]	Protein/Creatinine Ratio [g Protein/g Creatinine]	CRP [mg/L]
**Total INS** **N = 51**	1.90 (1.05–2.55)	363.0 (268.0–475.0)	6.2 (3.0–10.6)	2.90 (0.80–3.60)
**Group IA** **N = 20**	1.70 (1.40–2.20)	372.0 (297.0–464.0)	4.85 (2.40–7.90)	1.75 (0.40–3.60)
**Group IB** **N = 31**	2.40 (1.00–3.10)	329.5 (238.0–601.0)	7.0 (3.9–10.7)	3.10 (1.65–3.69)
**Group IIA** **N = 26**	1.90 (1.10–2.50)	366.5 (286.5–442.5)	4.71 (2.5–7.76) ^a^	3.30 (1.40–3.60)
**Group IIB** **N = 22**	2.0 (1.00–2.50)	329.5 (264.0–636.0)	9.6 (6.2–19.2)	2.56 (1.60–4.20)

**^a^**—group IIA versus group IIB, *p* = 0.023, Kruskal–Wallis test, INS—idiopathic nephrotic syndrome; CRP, C-reactive protein; Group IA—children with the first episode of INS; Group IB—children with relapses of INS; Group IIA—children treated only with GC, glucocorticosteroids; Group IIB—children treated with GC and corticosteroid-sparing agents.

**Table 2:** sTNF-α and uTNF-α levels of the total INS group both in relapse and remission, compared to the control.

	Groups		
Parameters	Whole Group INS Relapse N = 51	Whole Group INS Remission N = 35	Control N = 18
**sTNF-α** **[pg/mL]**	45.4 (43.8–47.4) ^a,b^	17.5 (15.6–18.8) ^a^	13.2 (12.6–13.6)
**uTNF-α** **[pg/mL]**	6.24 (5.50–7.10) ^a,b^	2.06 (1.81–2.56) ^a^	1.28 (1.23–1.33)

^a^—relapse INS versus control groups and remission INS versus control group (sTNF-α, uTNF-α), *p* < 0.001, Kruskal–Wallis test; ^b^—relapse INS versus remission INS (sTNF-α, uTNF-α), *p* < 0.001, Wilcoxon test. TNF-α—tumor necrosis factor alpha.

**Table 3:** sTNF-α and uTNF-α levels in INS subgroups, according to the number of relapses (group IA—the onset of disease, IB—subsequent relapses).

	Group			
Parameter	IA Relapse N = 20	IB Relapse N = 31	IA Remission N = 9	IB Remission N = 26
**sTNF-á** **[pg/mL]**	45.1 (43.7–46.8)	45.6 (43.9–48.2)	15.2 ^a^ (13.9–17.3)	17.7 (16.1–19.1)
**uTNF-á** **[pg/mL]**	6.27 (5.84–6.73)	6.24 (5.41–7.10)	2.55 (2.06–2.55)	2.06 (1.81–2.56)

^a^—group IA vs. IB, *p* < 0.013, Kruskal–Wallis test

**Table 4:** sTNF-α and uTNF-α levels in INS subgroups, according to the treatment modality.

	Groups			
Parameters	IIA Relapse N = 26	IIB Relapse N = 22	IIA Remission N = 17	IIB Remision N = 17
**sTNF-α** **[pg/mL]**	44.6 (43.6–46.8)	46.0 (44.8–48.4)	17.5 (15.6–19.1)	17.5 (15.9–18.4)
**uTNF-α** **[pg/mL]**	6.32 (5.50–7.10)	6.20 (5.50–6.67)	2.06 (1.56–2.55)	2.06 (1.92–2.56)

Kruskal–Wallis test.

## Data Availability

Data are contained within the article.
